# The Life and Times of Thomas Wakley

**Published:** 1898-12

**Authors:** 


**The Life and Times of Thomas Wakley.** By S. SQUIRE SPRIGGE, M.B. Pp. xix., 509. London: Longmans, Green and Co. 1897.

It is a remarkable thing that in these days when biographies are so much in vogue we should have had to wait thirty-six years for one of Thomas Wakley, the man who of all others the members of our own profession have to thank for the position, such as it is, they now occupy. But, perhaps, if Wakley's life had been written earlier we should not have had such a clear, judicious, and impartial account as that now edited by Dr. Sprigge; for the hatred and opposition that Wakley enjoyed during his lifetime might have in some degree influenced the writer to either over-state the value of work he did or to minimise the reforms effected. Wakley was not a gentle reformer: fearless in his criticism, and it must be confessed often injudicious in the violence of his language; he hit hard in defence of his arguments, but he had the right behind him, and in the end always carried his way.

The profession to-day owes a great debt of gratitude to Thomas Wakley. Brought up as a younger son in a Dorsetshire village, he found his advance in his profession crippled by the favouritism and nepotism that existed at the London hospitals; and to stamp out this he started the pioneer of weekly medical literature, the *Lancet*. The foundation of this paper was a bold venture for a young man, only known as the unfortunate victim of an unfounded scandal. But with a commendable energy and boldness he at once struck at the very root of the corrupt hospital system, and though more than once he suffered reverses, in the end he conquered.

It would take too long to recount all Wakley's achievements: but when we state that the Medical Act, the Anatomy Act, the Adulteration Act, the Medical Witnesses Act, were all largely due to him; that he effected the reform of the London hospitals, provided a good education for medical students, and made reform of the College of Surgeons a necessity, we are giving the main points of his life's work.

In his capacity as Member of Parliament, Editor of the *Lancet*, or Coroner, he acted fearlessly, unceasingly, and to the utmost of his power to do what he considered right, and to abolish what in his opinion was wrong; and though, as Dr. Sprigge says, the present constitution of the College of Surgeons does not satisfy its Members, or the General Medical Council command the respect of the general body of practitioners at the present day, they are far better than the condition of affairs previously existing.

We congratulate Dr. Sprigge on the very excellent manner in which he has worked up the immense amount of material required for this book, and also on the readable form in which he has presented it to the public. The life of Wakley should be in the hands of every medical man; for from it he may learn how much we owe to the first editor of the *Lancet*.

